# Effect of hyperbaric oxygen on mesenchymal stem cells for lumbar fusion *in vivo*

**DOI:** 10.1186/1471-2474-11-52

**Published:** 2010-03-19

**Authors:** Tsai-Sheng Fu, Steve WN Ueng, Tsung-Ting Tsai, Lih-Huei Chen, Song-Shu Lin, Wen-Jer Chen

**Affiliations:** 1 Department of Orthopaedic Surgery, Chang Gung Memorial Hospital, Chang Gung University School of Medicine, Kweishan, Taoyuan, Taiwan

## Abstract

**Background:**

Hyperbaric oxygen (HBO) therapy has been proved in improving bone healing, but its effects on mesenchymal stem cells (MSCs) *in vivo *is not clear. The aims of this study are to clarify whether the HBO therapy has the same enhancing effect on MSCs with regard to bone formation and maturation and to ascertain whether the transplanted MSCs survive in the grafted area and contribute to new bone formation.

**Methods:**

Twenty-three adult rabbits underwent posterolateral fusion at L4-L5 level. The animals were divided into three groups according to the material implanted and subsequent treatment: (1) Alginate carrier (n = 6); (2) Alginate-MSCs composite (n = 11); and (3) Alginate-MSCs composite with HBO therapy (n = 6). After 12 weeks, spine fusion was examined using radiographic examination, manual testing, and histological examination. Using a PKH fluorescence labeling system, whether the transplanted MSCs survived and contributed to new bone formation in the grafted area after HBO therapy was also examined.

**Results:**

The bilateral fusion areas in each animal were evaluated independently. By radiographic examination and manual palpation, union for the Alginate, Alginate-MSCs, and Alginate-MSCs-HBO groups was 0 of 12, 10 of 22, and 6 of 12 respectively. The difference between the Alginate-MSCs and Alginate-MSCs-HBO groups was not significant (P = 0.7997). The fluorescence microscopy histological analysis indicated that the transplanted PKH67-labeled MSCs survived and partly contributed to new bone formation in the grafted area.

**Conclusions:**

This study demonstrated that the preconditioned MSCs could survive and yield bone formation in the grafted area. HBO therapy did not enhance the osteogenic ability of MSCs and improve the success of spine fusion in the rabbit model. Although there was no significant effect of HBO therapy on MSCs for spine fusion, the study encourages us to research a more basic approach for determining the optimal oxygen tension and pressure that are required to maintain and enhance the osteogenic ability of preconditioned MSCs. Further controlled *in vivo *and *in vitro *studies are required for achieving a better understanding of the effect of HBO treatment on MSCs.

## Background

For posterolateral lumbar fusion, decorticated transverse processes are generally overlaid with bone graft materials. In recent decades, investigators have described various techniques for isolating human and animal mesenchymal stem cells (MSCs) from the bone marrow [1-3]. Culturing MSCs with dexamethasone, ascorbic acid, and β-glycerophosphate directs the cells into the osteogenic lineage [1,3]. In authors' pilot study, the alginate-MSCs (2 × 10^6 ^cells) composite successfully formed a bony mass between the transverse processes in a rabbit model. However, manual palpation revealed that the consistency of the bony mass was not solid enough [4].

When alginate-MSCs were laid over the transverse processes, the transplanted MSCs were exposed to a temporary avascular environment and oxygen deprivation. This condition may lead to the cell death or functional impairment, which can reduce the bone-forming ability of these cells; however, the precise effects of oxygen tension on MSCs have not been clearly established. Some studies have shown that hypoxia had a negative impact on cell growth and differentiation, while others had a positive effect on cell proliferation and osteoblastic differentiation [5-8]. Potier et al. recently reported that transplanted MSCs subjected to hypoxia in vivo exhibit a limited angiogenic factor secretion and persistent down-regulation of several osteoblastic markers, which may affect their bone-forming potential of these cells [9].

Several studies have proved the efficacy of hyperbaric oxygen (HBO) therapy in improving bone healing or bone formation. Penttinen et al. found in their study that the intermittent exposure of rats to HBO for 2 h daily at 2.5 atm resulted in hypertrophy of the cartilage and an increase in the rate of bone formation [10]. In our previous animal studies, HBO therapy improved bone healing of tibial lengthening [11,12] and the success of posterolateral fusion using autogenous iliac bone graft [13]. Therefore, it would be useful to study the effects of the HBO therapy on the transplanted MSCs for bone formation. The purposes of this study were to (1) document whether the HBO therapy has the same enhancing effect on MSCs with regard to bone formation and maturation; (2) ascertain whether the transplanted MSCs survive in the grafted area and contribute to new bone formation.

## Methods

In this study, 27 male New Zealand rabbits, weighing 3.5 to 4 kg, were used. Approval was obtained from the Institutional Animal Care and Use Committee at the institution of the authors prior to the study.

### Preparation of MSCs

The methods used for isolation and cultivation of the rabbit MSCs are as described in our previous study [14]. The rabbits were anesthetized with an intravenous injection of 5 mL of ketamine hydrochloride (Ketalar, Parke Davis, Taiwan) and Rompum (Bayer, Leverkusen, Germany) mixture, 10 mL of bone marrow was aspirated from the iliac crest under sterile conditions and was collected into a syringe containing 6000 units of heparin. The marrow sample was washed with Dulbecco's phosphate-buffered saline (DPBS), and disaggregated by gently passing the sample through a 21-gauge catheter and syringe to obtain a single-cell suspension. Cells were recovered after centrifugation at 600 × g for 10 min. Up to 2 × 10^8 ^nucleated cells in 5 mL DPBS were loaded onto a 25 mL Percoll cushion (Pharmacia Biotech) with a density of 1.073 g/mL in a 50 mL conical tube. Cells were separated by centrifugation at 1,100 × g for 40 min at 20°C. Nucleated cells were collected from the interface, diluted with two volumes of DPBS, and centrifuged at 900 × g. The cells were then resuspended, counted, and plated at 2 × 10^5 ^cells/cm^2 ^in T-75 flasks (Falcon). The cells were maintained in Dulbecco's Modified Eagle's Medium-Low Glucose (DMEM-LG; Gibco) containing 10% fetal bovine serum (FBS) and antibiotics (100 units/mL penicillin mixed with 100 μg/mL streptomycin; Gibco) at 37°C in a humidified atmosphere of 5% CO2 and 95% air. After 4 days of primary culturing, nonadherent cells were removed by changing the medium; medium was changed every 3 days thereafter. The MSCs, which grew as symmetrical colonies, were subcultured at 10-14 days with 0.05% trypsin and 0.53 mM EDTA for 5 min, rinsed from the substrate with serum-containing medium, collected by centrifugation at 800 × g for 5 min, and seeded into fresh flasks at 5,000-6,000 cells/cm^2^. Cultures were incubated in a humidified atmosphere of 5% CO2 and 95% air until cell confluence was achieved.

### Preparation of MSC loaded Alginate (Alginate-MSCs)

Cells were maintained in primary culture until confluent. Calcium sodium alginate (4.7 cm × 4.7 cm) (Kaltostat, ConvaTec, UK) was used as a carrier for mesenchymal stem cells. Approximately 2 × 10^6 ^MSCs were seeded on calcium sodium alginate. The Alginate-MSCs composite was maintained in a 1:1 mixture of the osteogenic medium (DMEM-LG containing 10% FBS, antibiotics, 100 μM ascorbate-2-phosphate, 10^-7 ^M dexamethasone, and 10 mM β-glycerophosphate) and incubated at 37°C in a humidified atmosphere of 5% CO_2 _and 95% air for one additional week. The medium was refreshed every 4 days.

### Staining with PKH membrane linkers and preparation of PKH67-labeled MSC loaded Alginate

The rabbit MSCs were isolated, cultured and labeled with PKH67 membrane linkers (0.5-10 μM, Sigma Chemical Company, St. Louis, MO). Alginate carriers were prepared for PKH67 membrane linker-labeled MSCs. Fluorescent labeling of the MSCs was performed by incubating 10^7 ^cells in each 1 mL Diluent C containing freshly prepared PKH67 membrane linker (0.5-10 μM, Sigma) for 10 min at room temperature. The cells were collected by centrifugation (400 × g, 10 min, 4°C), resuspended in Hanks' balanced salt solution (HBSS) supplemented with 10% FBS, and washed twice with HBSS. By using this procedure, 80-90% viable cells on an average were recovered, as determined by trypan blue exclusion testing. Approximately 2 × 10^6 ^PKH67-labeled MSCs were seeded on calcium alginate (4.7 cm × 4.7 cm) (Kaltostat, ConvaTec, UK). The Alginate-PKH67-labeled MSCs composite was then maintained in a 1:1 mixture of the osteogenic medium and incubated under conditions identical to those used for the preparation of the Alginate-MSCs composite.

### Animal experiment

The rabbit was anaesthetized via an intravenous injection of 5 mL of ketamine hydrochloride (Ketalar, Parke Davis, Taiwan) mixed with Rompum (Bayer, Leverkusen, Germany), the rabbit was then shaved and prepared with betadine. Under aseptic conditions, a 10-cm dorsal midline skin incision was made, followed by 2 paramedian fascial incisions to expose the L4 and L5 transverse processes. After decortication of the transverse processes with an air tome, the Alginate or Alginate-MSCs composite was deposited between the transverse processes. The fascia and the skin incisions were then sutured. The animals were divided into three groups according to the material implanted and subsequent treatment: (1) Alginate carrier only; (2) Alginate-MSCs composite; and (3) Alginate-MSCs composite with HBO therapy. The rabbits in the HBO therapy group were treated with 100% oxygen at 2.5 atm for 2 h daily, 5 times a week, while those in the other groups were maintained under normal room air. At the end of 12 weeks, the rabbits were sacrificed for evaluation.

### Radiographic analysis

All the animals underwent posteroanterior radiographs and 2 mm thin-cut computed tomography (CT) scan of the lumbosacral spine. The bilateral fusion areas in each animal were evaluated independently. Successful radiographic union was determined as an uninterrupted bone bridge between the intertransverse processes. The CT scans were also evaluated to identify the presence of bone formation in any unintended areas including the spinal canal or ventral to the intertransverse process membrane.

### Manual palpation of spine fusion

Following radiography, L3-L6 was excised *en block *from the animals. The fusion areas were manipulated with sufficient force to evaluate the presence of any motion and avoid causing gross trauma. Only the fusion mass that was identified as having no gross motion was considered as successful solid union.

### Histological evaluation

The L4-L5 fusion segment was fixed in 10% neutral buffered formaldehyde, decalcified, dehydrated through alcohol gradients, cleared and embedded in paraffin blocks. Tissue blocks were sectioned in the sagittal plane of the fusion mass included the transverse processes at 5 μm and stained with hematoxylin and eosin and Masson trichrome methods. New bone formation, the interface between the newly formed bone and the original transverse process, and any inflammatory response to the carriers were subsequently assessed.

### In situ PKH67-labeled MSCs tracing

In order to ascertain the survival of the transplanted MSCs, the PKH67-labeled MSCs in the recipients were directly observed. Specimens harvested from the L4-L5 fusion area were fixed in 2% paraformaldehyde, decalcified, and embedded in Tissue-Tek O.C.T. compound. Cryosections (8 μm) were sliced from the center of the specimens. An Olympus fluorescence microscope was used to directly examine PKH67-labeled MSCs in the recipient specimens. RGB image reconstruction was performed under green fluorescence under green fluorescence of PKH67-labeled cells.

### Statistical analysis

The fusion status of the experimental animals was then assessed. Differences between the Alginate-MSCs and Alginate-MSCs-HBO groups were analyzed using chi-square test with a significant difference assumed at P < 0.05.

## Results

Posterolateral intertransverse fusion was performed in a total of 27 experimental rabbits. The Alginate carrier only group and Alinate-MSCs group comprised 6 and 11 animals that were extremely tolerant to the surgical procedures and exhibited no perioperative complications. Ten rabbits received HBO therapy. Four animals developed complications and died due to oxygen intoxication; of these, 1 developed complications during 5^th ^HBO treatment; 2, during the 20^th ^HBO treatment; and 1, during the 30^th ^HBO treatment. Finally, 6 animals that underwent 60 rounds of HBO treatments were included in this study.

### Radiographic analysis

**Figure 1:**
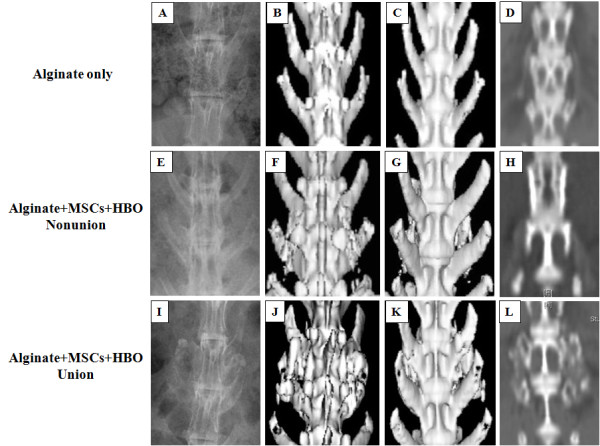
**Results of plain radiographs (A, E, I), dorsal view (B, F, J) and ventral view (C, G, K) 3D CT scan images, and coronary reconstructive images (D, H, L) in groups of Alginate only and Alginate-MSCs under hyperbaric oxygen (HBO) treatment**. **A, B, C, D**. The alginate carriers were completely absorbed and there was no bone formation between the transverse processes in the animal implanted with alginate without mesenchymal stem cells. **E, F, G, H**. Partial bone formation between the transverse processes in the animal implanted with Alginate-MSCs after HBO therapy. The scattered and uncalcified bone mass formation without successful fusion was noted. **I, J, K, L**. A successful, continuous bone fusion mass with calcification was noted between the transverse processes in the animal implanted with Alginate-MSCs after HBO therapy. No new bone formation occurred in any unintended areas on the CT scan images.

**Figure 2:**
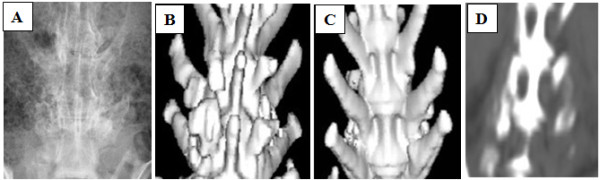
**Results of plain radiographs (A), dorsal view (B) and ventral view (C) 3D CT scan images, and coronary reconstructive images (D) in groups of Alginate-MSCs without HBO treatment**. A successful, continuous bone fusion mass with calcification was noted between the left intertransverse space. On the contrary, a scattered and uncalcified bone mass without fusion was noted between the right intertransverse space.

There were 12 fusion areas in the Alginate group, 22 in the Alginate-MSCs group, and 12 in the Alginate-MSCs-HBO group, respectively. On plain radiographs and CT scan images, there was not any bone formation observed between the transverse processes in the animals of Alginate group (Figure 1A, B, C, D). However, 10 of the 22 fusion areas in the Alginate-MSCs group were considered as radiographic union compared with 6 of the 12 fusion areas in the Alginate-MSCs-HBO group (Figure 1I, J, K, L) (P = 0.7997). In the other animals of both groups that no continuous fusion mass formation was observed on the plain radiographs, the CT scan images revealed scattered bone mass formation between the transverse processes (Figure 1E, F, G, H and Figure 2). No new bone formation occurred in any unintended areas on the CT scan images.

### Manual palpation

In the six specimens of Alginate group, the alginate carriers were absorbed and there was not any bone formation found. In the other hand, the remnants of the alginate-MSCs composite were still present in the specimens of Alginate-MSCs group and the Alginate-MSCs-HBO group. Manual palpation revealed that a slight motion between the transverse processes in any animals considered as radiographic union in both groups.

### Histological evaluation

**Figure 3:**
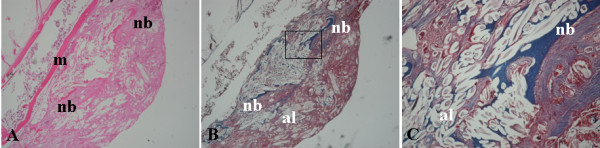
**The histological sections of the graft in the animals implanted with Alginate-MSCs composite**. **A**. H&E stains showed new bone formation was present in the graft fragments and there was no evidence of inflammatory cells or reactions to the alginate carrier (original magnification ×4). **B, C**. Masson trichrome stains showed new trabecular bone with collagen-fibril formation (blue areas) in the graft (B, original magnification ×4; C, original magnification ×10). m = muscle; nb = new bone; al = alginate remnants.

In the animal specimens from both the Alginate-MSCs group and the Alginate-MSCs-HBO groups, the alginate had disintegrated into minute remnants. Bone formation was present in the graft fragments. There was no evidence of inflammatory cells or reactions to the alginate carrier (Figure 3). The specimens of radiographic union in both groups exhibited bone formation in the intertransverse interval, but not interconnected with the recipient transverse process. The results closely matched the results of manual palpation.

### Histological evaluation for tracing of PKH67-labeled MSCs in the recipients

**Figure 4:**
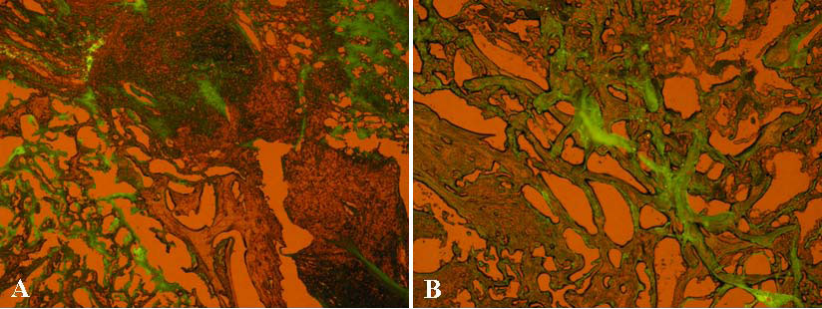
**The histological sections of the graft in the animals implanted with PKH67-labeled MSCs after hyperbaric oxygen therapy**. **A, B**. The examination revealed green fluorescent spots in the grafted area (A, original magnification ×10; B, original magnification ×20). It indicated that the transplanted MSCs were viable and partly contributed to new bone formation in the grafted area.

PKH67-labeled MSCs were implanted in 4 animals, of which 2 belonged to the Alginate-MSCs group and 2 to the Alginate-MSCs-HBO group. In all 4 animals, bone mass formation was observed on the CT scan images although it was not continuous. The labeled cells were assessed by fluorescence microscopic examination and revealed green fluorescent spots in the grafted area (Figure 4). The findings indicated that the transplanted MSCs had survived and partly contributed to new bone formation in the grafted area.

## Discussion

Several studies have proved the efficacy of HBO therapy in improving bone healing or bone formation [10-13], but the effects HBO therapy on mesenchymal stem cells *in vivo *is not clear. This study attempted to (1) clarify whether the HBO therapy has the same enhancing effect on MSCs with regard to bone formation and maturation; (2) ascertain whether the transplanted MSCs survive in the grafted area and contribute to new bone formation. Using a PKH fluorescence labeling system, it indicated that the transplanted MSCs survived in the grafted area and contributed to new bone formation. However, the final results of the present study revealed that HBO treatment did not enhance the preconditioned MSCs with regard to the success of posterolateral lumbar fusion in a validated rabbit model.

After bone grafting, the formation of new bone is closely associated with the rate of vascularization [15]. In the posterolateral fusion area, the major blood supply for vascularization of the bone graft was from the upper and lower transverse processes [16]. After dissection, the healing environment between the transverse processes is relatively hypovascular. This hypovascular environment can result in cell death or functional impairment of the implanted MSCs, which ultimately affects their bone-forming ability. HBO is known to accelerate the development of new blood vessels in hypovascular tissue, which increases vascularization in the operated area and enhances bone healing. In the present study, the reason why HBO did not enhance the osteogenic potency of the preconditioned MSCs is not quite clear. It is known that stem cells residing in the bone marrow are similarly exposed to low oxygen tension [17]. However, the precise effects of oxygen tension on osteoprogenitor or osteoblast-like cells have not been clearly established. Some studies have shown that hypoxia has a negative impact on cell growth and differentiation, whereas others have demonstrated a positive effect of hypoxia on cell proliferation and osteoblastic differentiation [5-9]. In the literatures, no published data is available with regard to the effect of HBO on MSCs. In the present study, the results of the histological examination revealed the presence of PKH67-labeled MSCs in the grafted area. Although the number of cells was not counted, it indicated that the implanted MSCs were kept in the alginate carrier and survived even after the HBO therapy. We supposed that the phenotype and osteogenic potency of the preconditioned MSCs might be altered after HBO therapy. It might be the reason that the HBO therapy did not have enhancing effect on the bone-forming potential of MSCs. Further basic studies with regard to the optimal oxygen tension and pressure required for the maintenance and enhancement of the osteogenic ability of preconditioned MSCs need to be carried out.

In order to ascertain the survival of the transplanted MSCs and to determine whether the new bone had been derived from the transplanted MSCs, we used the PKH-67 dye, which was incorporated into cell membranes and was equally distributed to the daughter cells during division. The dye is a nonradioactive and noncytotoxic substance and possesses a fluorescence half-life of >100 days in erythrocytes or MSCs [18]. In the current study, bone mass formation was not continuous and solid in all 4 animals transplanted with PKH67-labeled MSCs. We did think that the PKH-67 dye would influence the osteogenic ability of MSCs for spine fusion. However, the fluorescence microscopic examination revealed green fluorescent spots in the grafted area (Figure 4). The findings indicated that the transplanted MSCs had survived and partly contributed to new bone formation in the grafted area.

The optimal number of MSCs that should be implanted for achieving successful fusion is a critical parameter. Minamide et al. reported that implantation of 1 million bone marrow cells resulted in a poor fusion rate and that 100 million cells are required to be implanted for successful posterior spinal fusion in a rabbit model [19]. Nakajima et al. reported that implantation of 1.5 million preconditioned cells induced bone formation and achieved successful posterolateral fusion in 4 of 5 rabbits. In contrast, only 2 of 6 rabbits achieved successful fusion when the implanted cells were not cultured in the osteogenic differentiation medium [20]. In the current study, we selected 2 million cells on the basis of the results of our previous study. The data showed that such a number of preconditioned MSCs could induce bone formation between the intertransverse spaces and a low dose (2.5 μg) of recombinant human bone morphogenetic protein-2 (rhBMP-2) could enhance the osteogenic potency of MSCs and improves fusion success [4]. In the current study, we attempted to find some other methods to improve the osteogenic potency of MSCs. It remains unclear whether better results will be obtained if a larger number of cells that have been stimulated with the HBO are used; however, these findings deserve further investigation.

A limitation of this study is the insufficient number of animals that comprised in the HBO treatment group to a powerful conclusion on the no significant effect of HBO therapy on MSCs for spine fusion. Due to the high mortality rate (4 of the 10 animal died) during the treatment course, we decided not to perform the HBO therapy on any more animals. This is the reason why only 6 animals comprised the HBO group. This study, however, does not discuss the potential risks of the HBO therapy and only addresses the gross effect of the HBO therapy on the preconditioned MSCs for posterolateral fusion. It may, therefore, be of great interest to determine the effects of the HBO therapy on the osteogenic potential in MSCs.

## Conclusions

This study demonstrated that the preconditioned MSCs could survive and yield bone formation in the grafted area. HBO therapy did not enhance the osteogenic ability of MSCs and improve the success of spine fusion in the rabbit model. Although there was no significant effect of HBO therapy on MSCs for spine fusion, the study encourages us to research a more basic approach for determining the optimal oxygen tension and pressure that are required to maintain and enhance the osteogenic ability of preconditioned MSCs. Further controlled *in vivo *and *in vitro *studies are required for achieving a better understanding of the effect of HBO treatment on MSCs.

## Competing interests

The authors declare that they have no competing interests.

## Authors' contributions

The following authors have: (1) designed the study: WJC, SWNU, TSF; (2) carried out all experiments and gathered the data: TSF, TTT, SSL; (3) analyzed the data: WJC, SWNU, LHC, TTT, SSL, TSF; (4) written the initial draft: TSF, TTT, SSL; (5) ensured the accuracy of the data and analysis: WJC, SWNU, LHC. All authors have read and approved the final manuscript.

## Pre-publication history

The pre-publication history for this paper can be accessed here:

http://www.biomedcentral.com/1471-2474/11/52/prepub
